# Sustained Vaccination Coverage during the Coronavirus Disease 2019 Epidemic in the Republic of Korea

**DOI:** 10.3390/vaccines9010002

**Published:** 2020-12-22

**Authors:** Jeong Hee Yu, Hang jin Jeong, Seon Ju Kim, Jae Young Lee, Young June Choe, Eun Hwa Choi, En Hi Cho

**Affiliations:** 1 Division of National Immunization Program and Vaccine-Preventable Disease Control, Korea Disease Control and Prevention Agency, Cheongju 28159, Korea; cheeyu@korea.kr (J.H.Y.); jhjin9035@korea.kr (H.j.J.); ektjtwkd@korea.kr (S.J.K.); pgzdtk198@korea.kr (J.Y.L.); 2 Department of Social and Preventive Medicine, Hallym University College of Medicine, Chuncheon 24252, Korea; ychoe@hallym.ac.kr or; 3 Department of Pediatrics, Seoul National University College of Medicine, Seoul 03080, Korea; eunchoi@snu.ac.kr

**Keywords:** SARS-CoV-2, coronavirus, COVID-19, vaccination, vaccine coverage, vaccine-preventable disease

## Abstract

Amid the COVID-19 pandemic, vaccination coverage may decline due to limited accessibility to healthcare. We assessed the impact of the COVID-19 pandemic on vaccination coverage and the incidence of vaccine-preventable diseases (VPDs) in the Republic of Korea. National vaccination coverage of 10 essential vaccines administered to children between January–June 2019 and January–June 2020 was analyzed. The national incidence of selected VPDs was compared for the corresponding periods. During the COVID-19 outbreak, the vaccination rate in children aged 0–35 months in Korea did not decrease significantly, whereas the vaccination rate for children aged 4–6 years decreased by 1.4–1.9%. The overall incidence of VPDs decreased by 10–50% between 2019 and 2020, especially with varicella. Thus, the COVID-19 pandemic did not result in a decrease in vaccination coverage among Korean children, which prevented a surge in VPD incidence. Maintaining essential vaccination coverage without interruption is important during the response to the COVID-19 pandemic.

## 1. Introduction

Vaccination is one of the most effective ways to prevent and control vaccine-preventable diseases (VPDs). The management of vaccine-preventable disease (VPD) during the coronavirus disease (COVID-19) pandemic is a challenge in many countries due to various reasons, from inaccessibility to healthcare to a surge in capacity. Nonetheless, if immunization services are not sustained, there is likely to be an increase in the incidence of VPDs, as seen in the Ebola outbreak in West African countries [1]. To prevent a simultaneous epidemic of COVID-19 and VPD and to prevent outbreaks of VPD after the COVID-19 pandemic has passed, operation of the vaccination system should be continued without interruption [2]. In 2020, private clinics and public sectors were informed additionally on safe vaccination practice by adherence to infection control and prevention policy within the primary care sites in South Korea.

Herein, we aimed to compare the national vaccination coverage in Korea in the period from January to June 2020 during the COVID-19 pandemic with that in the same period in 2019.

## 2. Materials and Methods

**Figure 1:**
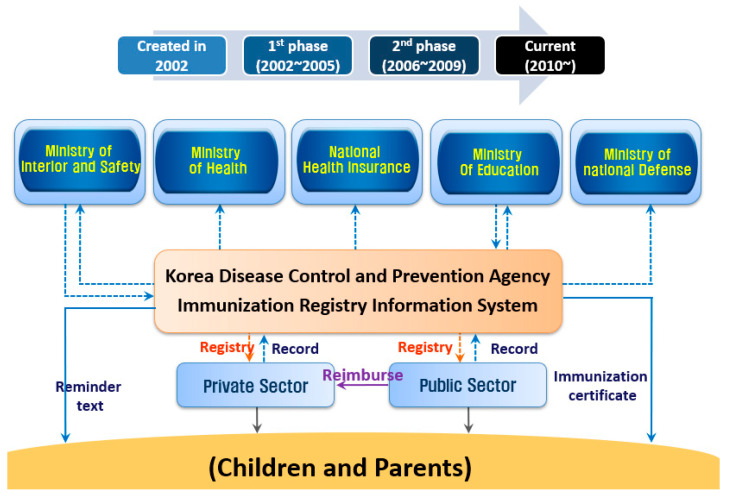
Scheme for Immunization Registry Information System (IRIS) in Korean National Immunization Program (NIP).

Vaccination coverage was calculated based on the resident registration population data of the Korean Ministry of Public Administration and Security [3]. The immunization registry covers 100% of national immunization provided in the country, and therefore is not a sampled estimate of coverage. Vaccination record data were obtained from the computerized registration data of the integrated vaccination management system of the Korea Disease Control and Prevention Agency (KDCA) [4]. The KDCA receives the vaccination data of the National Immunization Program (NIP) electronically from public health centers and private medical institutions across the country (Figure 1). We used vaccination data for children under the age of 6 years enrolled in NIP from January to June 2019 and January to June 2020. Vaccination records were extracted on 14 August 2020.

The vaccination coverage was calculated as the proportion of vaccinated individuals who completed vaccination according to the standard vaccination schedule. Bacillus Calmette-Guérin (BCG) vaccine coverage was calculated as the proportion of children who completed the vaccination by 1 month of age, and hepatitis B (HepB) vaccination coverage was calculated as the proportion of children who completed the first vaccination within 24 h of birth and completed the 2nd and 3rd vaccinations between 1 and 6 months of age, respectively. Diphtheria-tetanus-acellular pertussis (DTaP) vaccine coverage was calculated as the proportion of children who completed 3 basic vaccinations at 2, 4, and 6 months of age and the 2 booster vaccinations between 15 and 18 months and 4 and 6 years of age. Inactivated polio vaccine (IPV) coverage was calculated as the proportion of children who completed the basic vaccination at 2, 4, and 6 months of age and completed the booster vaccination between 4 and 6 years of age. *Haemophilus influenzae* type b vaccine (Hib) and pneumococcal conjugate vaccine (PCV) vaccination coverage were calculated as the proportion of children who completed the basic vaccination at 2, 4, and 6 months of age and the proportion of children who completed booster doses between 12 and 15 months of age. Measles-mumps-rubella (MMR) vaccine coverage was calculated as the proportion of children who completed the second vaccination between 4 and 6 years of age after a first vaccination between 12 and 15 months of age. Varicella (VAR) vaccination coverage was calculated as the percentage of children who completed the first vaccination between 12 and 15 months of age. Hepatitis A (HepA) vaccination coverage was calculated as the proportion of children who completed the second dose at least 6 months after the first dose and between 12 and 23 months of age. Japanese encephalitis (JE) vaccination coverage was calculated as the proportion of children who completed the 2nd vaccination between 12 and 23 months of age, received the 3rd vaccination 12 months later, and received 1 additional vaccination before the age of 6 years. The 23-valent pneumococcal polysaccharide vaccine coverage was calculated as the proportion of adults aged 65 years who received a single dose of 23-valent vaccine during the study period.

The incidence of hepatitis B, pertussis, tetanus, Hib type b invasive disease, invasive pneumococcal disease, measles, mumps, and rubella during the periods January to June 2018 and January to June 2020 was compared using the national notifiable disease surveillance data reported to the Korea Disease Control and Prevention Agency (KDCA).

This study only used aggregated data that did not identify individuals; as per Korean legislation and rules, it did not require ethical review and approval. The requirement for informed consent did not apply.

## 3. Results

The vaccination coverage in 2020 was generally sustained at a comparable level as in 2019 (Table 1). The vaccination rate for one BCG vaccination in the prior 12 months, HepB1–HepB3, DTaP1–DTap3, IPV1–IPV3, Hib1–Hib3, and PCV1–PCV3 increased by 1–1.3% in 2020. Of the vaccines administered before the age of 3 years, the vaccination coverage of the VAR, MMR, and HepA vaccines starting one year after birth decreased by 0.1–0.3%. The age group with the largest reduction in vaccination coverage was children aged 4–6 years, among whom vaccination coverage decreased by 1.4–1.9%.

**Figure 2:**
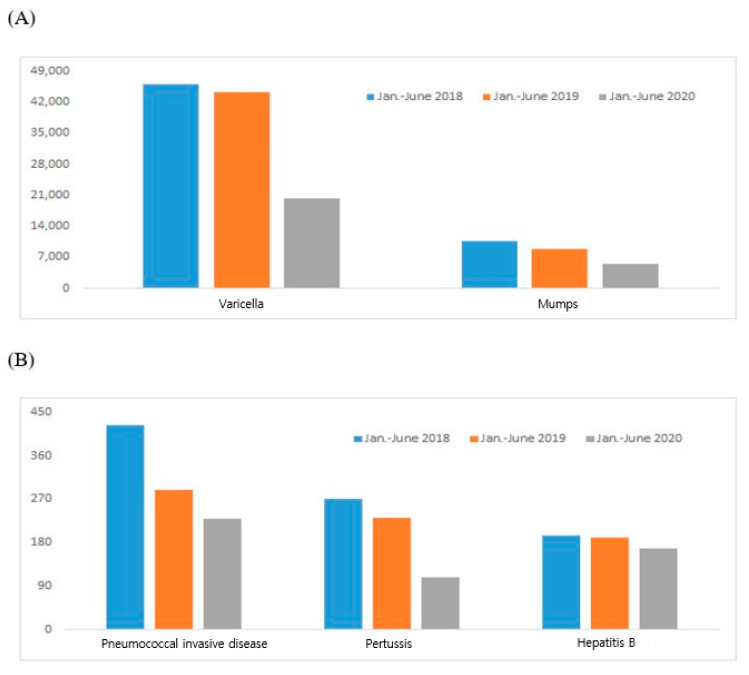
Vaccine-preventable disease notifications (2018–2020). The incidence of the most vaccine-preventable diseases decreased in January to June 2020 during the COVID-19 pandemic. (**A**) Varicella and mumps (**B**) invasive pneumococcal infections, pertussis, hepatitis B.

The incidence of varicella and mumps, which occur mainly in schools between March and April at the beginning of the school year, each decreased by 50% in 2020 (Figure 2A). Invasive pneumococcal infection, pertussis, and hepatitis B incidence decreased by approximately 12–40% in 2020 (Figure 2B). The incidence of tetanus, which averages <20 cases per year, and the incidence of Hib and rubella, which average 2 to 4 cases per year, were similar in the first half of 2019 and 2020 (Table 2). Measles has maintained an annual incidence of approximately 10 to 20 cases per year since 2015. In the first half of 2019, 169 cases were reported, but in half of 2020, 6 cases were reported, which is comparable to the incidence prior to 2019 (Table 2).

## 4. Discussion

In this study, we found sustained vaccination coverage in Korean children during the COVID-19 pandemic in the setting of stringent social distancing measures and with limited mass gathering events in the first half of 2020. This finding is largely due to the strong public vaccination program that had been in place. The Korean NIP is characterized by full free support for national vaccinations of children under 12 years of age and the management of the vaccination ability of the individual using an integrated vaccination management system [3]. The vaccination registration project has been implemented since 2002 to systematically manage individual vaccination records by developing and distributing a vaccination record registration system using information technology infrastructure and establishing a record management system [5]. In the early stages of development, only provision of vaccination service was provided, but now, the service’s issuance of vaccination certificates makes it easier to verify vaccination history, and the operation of an adverse reaction monitoring system after vaccination has been extended to ensure safe vaccination [6].

Another key to maintaining vaccination coverage has been the endeavor to ensure that children have access to convenient and safe vaccination services at minimal cost in Korea. The national vaccination program in Korea started providing free vaccination in the public sector in 1957, expanded to a pilot project to provide government support to cover essential vaccination costs by private medical institutions in 2009, and further expanded to provide government support for free vaccination in 2014 [7]. The expansion of government support has improved access so that individuals requiring vaccination can conveniently receive them at medical institutions close to their residence [8]. The budget for government support for vaccination by private sectors increased by 18 times from USD 14,977,000 in 2014 to USD 276,230,000 in 2020 [6]. The average vaccination coverage (97%) for children aged 3 years in Korea [9] is 2–10 percentage points higher than that in the United States [10], United Kingdom [11], and Australia [12]. Financial support for essential vaccines, the provision of advance/delayed notification information services for vaccination schedule management, and the systematic management of vaccination records by operating the vaccination computerized registration system are the main contributory factors to maintaining high vaccination coverage [8].

The social distancing measures in 2020 have not only impacted the immunization practice and coverage, but they have also affected the incidence of vaccine-preventable diseases that are transmissible between persons. The VPD incidence in the first half of 2020 decreased from 2019. Factors such as social distancing campaigns, wearing masks, hand washing, delays in schools opening to students, and online classes may have influenced the decrease in VPD incidence. A recent study found a modest decline in incidence for mumps and varicella, while little has changed for hepatitis B incidence. This difference may be attributable to the mode of transmission and affected age (adults rather than children) [13]. However, if vaccination is neglected due to complacency, the risk of resurgence of VPDs exists; therefore, vaccination should be performed without interruption. In crisis situations, such as the COVID-19 pandemic, it will be very difficult to continue immunization without interruption. However, if medical staff and vaccinees follow the quarantine rules and maintain vaccination without interruption, it is expected that they will be able to return to their daily routine sooner without additional measures for the epidemic of infectious diseases after the end of COVID-19. Health authorities should continuously inform the public of the importance of vaccination and guide them so that vaccination can be provided continuously, and the vaccinees should also follow the quarantine rules and endeavor to complete vaccination according to the standard schedule.

The World Health Organization issued a guideline recommending the maintenance of vaccinations on 26 March, foreseeing a possible decline in vaccination demand due to the COVID-19 pandemic [2]. The guidelines set vaccination priorities and described measures to ensure that vaccinations are performed under safe conditions and that if vaccination services are interrupted due to COVID-19, a catch-up vaccination strategy is established once the COVID-19 epidemic has ended. In this context, we have also implemented a policy to promote vaccination at private clinics. Considering the increasing number of vaccinations provided in private clinics, a guide for safe vaccination was created and released to private clinic staff and the public on 27 April 2020. In the guidebook, the items to be followed by the vaccinee or guardian and those to be followed by private clinics were organized separately. In the case of vaccinees or guardians, they are required to book in advance at a private clinic, answer a questionnaire, be limited to the minimum number of people required for the vaccination, and wear a mask. In the waiting room, a sufficient distance of approximately 2 m must be maintained, and hand sanitization must be thoroughly conducted when entering and exiting private clinics. Private clinics are required to adjust the number of visitors by making reservations in advance and to check visitors for fever. When vaccination is performed, medical staff are required to wear protective equipment, to separate the vaccination area from the treatment area, and to separate the path between vaccinees and patients with health problems to minimize contact.

Our findings have a number of limitations. Firstly, we used aggregated, deidentified data to perform an ecological study, which is limited because the measures of vaccine coverage and VPD incidences are only a proxy based on the average in the population. Caution is needed when applying grouped results to the individual level. Secondly, during the COVID-19 pandemic, data on reportable VPDs may not be representative in the setting when patients do not visit clinics/hospitals for medical care. Although mandated by law, we do not know the estimation for the reporting rate of nationally notifiable diseases. Despite these limitations, we believe that our data may represent the trend in countries with long-standing vaccination policies that resulted in changes in the epidemiological patterns of VPDs. Our observation may aid in establishing baseline data for future discussion regarding vaccination strategies and, moreover, may serve as a reference for other countries when they are planning vaccination policy in the setting of the COVID-19 pandemic.

As we have been in the COVID-19 pandemic for several months, there is a surge of cases in many parts of the world. Traditional pandemic preparedness plans address containment and mitigation strategy against novel pathogens. However, given the limited pharmaceutical intervention, maintaining the essential health services including immunization will affect the net public health safety afterwards.

In conclusion, we found that the COVID-19 outbreak did not significantly affect the childhood vaccination rate in Korea in the setting of strong accessibility to care conferring high routine vaccination coverage. It is important that every country makes special efforts to provide uninterrupted vaccination to protect public health amid the global COVID-19 pandemic.

## Figures and Tables

**Table 1:** Comparison of vaccination coverage in January to June 2019 and January to June 2020.

Age Group	Vaccine		January to June 2019	January to June 2020	Change (%)
			Coverage (%)	Coverage (%)	
6 mo	BCG		97.6	98.9	1.3
	HepB	1st	98.0	99.1	1.1
		2nd	98.0	99.1	1.1
		3rd	94.4	96.1	1.7
	DTaP	1st	97.8	98.9	1.1
		2nd	97.3	98.3	1.0
		3rd	92.7	94.1	1.4
	IPV	1st	97.8	98.9	1.1
		2nd	97.3	98.3	1.0
		3rd	92.6	94.1	1.5
	Hib	1st	97.8	98.9	1.1
		2nd	97.3	98.2	0.9
		3rd	92.4	93.9	1.5
	PCV	1st	97.7	98.9	1.2
		2nd	97.0	98.1	1.1
		3rd	90.0	92.4	2.4
12–15 mo	Hib	4th	89.3	90.8	1.5
	PCV	4th	88.3	89.9	1.6
	MMR	1st	95.7	95.5	−0.2
	Var		95.5	95.4	−0.1
15–18 mo	DTaP	4th	88.5	88.8	0.3
12–23 mo	HepA	1st	97.1	96.9	−0.2
	JE	1st	97.0	96.7	−0.3
		2nd	96.7	96.4	−0.3
24–35 mo	HepA	2nd	93.6	95.0	1.4
	JE	3rd	90.3	90.6	0.3
48–72 mo	DTaP	5th	95.4	93.5	−1.9
	IPV	4th	96.3	94.4	−1.9
	MMR	2nd	96.5	94.8	−1.7
	JE	4th	91.0	89.6	−1.4

**Table 2:** Number of vaccine-preventable disease notifications according to period.

Infectious Disease	Janurary–June 2018	Janurary–June 2019	Janurary–June 2020
Tetanus	16	17	15
*Haemophilus influenzae* type b	2	0	1
Rubella	0	4	2
Measles	11	169	6

## Data Availability

Please refer to suggested Data Availability Statements in section “MDPI Research Data Policies” at https://www.mdpi.com/ethics.

## References

[1] Masresha B.G., Luce R., Weldegebriel G., Katsande R., Gasasira A., Mihigo R. (2020). The impact of a prolonged ebola outbreak on measles elimination activities in Guinea, Liberia and Sierra Leone, 2014–2015. Pan Afr. Med. J..

[2] World Health Organization (2020). Guiding Principles for Immunization Activities during the COVID-19 Pandemic.

[3] Korea Centers for Disease Control and Prevention (KCDC) (2020). National Immunization Program Support Project Management Guidelines.

[4] Korea Centers for Disease Control and Prevention (KCDC) (2019). 2019–2020 Seasonal Influenza National Immunization Support Project Management Guidelines.

[5] Cha S.H. (2012). The history of vaccination and current vaccination policies in Korea. Clin. Exp. Vaccine Res..

[6] Korea Centers for Disease Control and Prevention (KCDC) (2020). White Paper on Disease Control and Prevention.

[7] Choe Y.J., Han O.P., Cho H., Bae G.R., Chun B.C., Kim J.H., Kim K.H., Lee H.J., Choi E.H. (2014). Prioritization of the introduction of new vaccines to the national immunization program in the Republic of Korea. Vaccine.

[8] Park B., Choi E.J., Park B., Han H., Cho S.J., Choi H.J., Lee S., Park H. (2018). Factors Influencing vaccination in Korea: Findings From Focus Group Interviews. J. Prev. Med. Public Health.

[9] Statics Korea (2020). National Immunization Rate in Korea, 2019.

[10] Hill H.A., Elam-Evans L.D., Yankey D., Singleton J.A., Kang Y. (2018). Vaccination Coverage among Children Aged 19–35 Months—United States, 2017. MMWR Morb. Mortal. Wkly. Rep..

[11] National Statistics (2019). Childhood Vaccination Coverage Statistics, England, 2018–2019.

[12] Hull B., Hendry A., Dey A., Brotherton J., Macartney K., Beard F. (2018). Annual Immunisation Coverage Report 2017. Commun. Dis. Intell..

[13] Yun H.E., Ryu B.Y., Choe Y.J. (2020). Impact of social distancing on incidence of vaccine-preventable diseases, South Korea. J. Med. Virol..

